# Adherence to the Canadian CT Head Rule in a Nova Scotian Emergency and Trauma Center

**DOI:** 10.7759/cureus.39484

**Published:** 2023-05-25

**Authors:** Amrit Sampalli, Jessie Kang, Sam G Campbell, Constance H LeBlanc

**Affiliations:** 1 Medicine, Dalhousie University, Halifax, CAN; 2 Diagnostic Radiology, Dalhousie University, Halifax, CAN; 3 Emergency Medicine, Dalhousie University, Halifax, CAN

**Keywords:** guideline adherence, ct scan head, head trauma, choosing wisely, continuous quality improvement

## Abstract

Background and aims

Choosing Wisely Nova Scotia (CWNS), an affiliate of Choosing Wisely Canada (CWC), aims to address unnecessary care and tests through literature-informed lists developed by various disciplines. CWC has identified unnecessary head CTs among the top five tests, procedures, and treatments to question within the emergency department setting. The Canadian CT-scan Head Rule (CCHR) has been found to be the most effective clinical decision rule in adults with minor head injuries. This study aimed to better understand the current status of CCHR use in Nova Scotia, we conducted a retrospective audit of patient charts at the Charles V. Keating Emergency and Trauma Center in Halifax, Nova Scotia.

Materials and methods

Our mixed methods design included a narrative literature review, a retrospective chart audit, and a qualitative audit-feedback component with physicians who work in the emergency department (ED). The chart audit applied the guidelines for adherence to the CCHR and reported on the level of compliance within the ED.

Results

Analysis of qualitative data is included here, in parallel with in-depth analysis to contextualize findings from the chart audit. A total of 302 charts of patients presenting to the surveyed site were retrospectively reviewed for this study. Of the 37 cases where the CT head was indicated as per the CCHR, a CT was ordered 32 (86.5%) times. Of the 176 cases where a CT head was not indicated as per the CCHR, a CT was not ordered 155 (88.1%) times. Therefore, the CCHR was followed in 187 (87.8%) of the total 213 cases where the CCHR should be applied.

Conclusions

Our review revealed that the CCHR was adhered in 87.8% of cases at the surveyed ED. Identifying contextual factors that facilitate or hinder the application of CCHR in practice is critical to achieving the goal of reducing unnecessary CTs. This work will be presented to the physician group to engage and understand factors that are enablers in the process of ED minor head injury care.

## Introduction

In 2017, the Canadian Institute for Health Information reported that as many as 30% of patients in Canada received care that was deemed unnecessary [1]. Combined with the fact that all investigations and interventions carry some degree of risk, physicians must strive to minimize harm and provide care that adds value to both the patient and the system. Choosing Wisely Nova Scotia (CWNS), an affiliate of Choosing Wisely Canada (CWC), aims to address unnecessary care using literature-informed lists developed by various disciplines.

Emergency department (ED) physicians are required to make time-sensitive decisions regarding their patient’s care daily. Studies of utilization of ED diagnostic tests have shown an increasing trend in the utilization of computer tomography (CT) in EDs without a clear link to improved patient outcomes [2,3]. CT examinations pose a set of inherent risks including ionizing radiation, intravenous contrast complications, or incidental findings that lead to further unnecessary tests. Unwarranted CT examinations can increase ED wait times, length of stay, and overall financial burden [4-6]. Finally, when testing is conducted in patients with very low pre-test probability, the post-test probability changes so marginally that no change in management results from having undertaken the test [7]. Approximately 60-75% of patients with minor head injuries undergo head CT in EDs [2]. Choosing Wisely Canada, in conjunction with the Canadian Association of Emergency Physicians (CAEP), has identified unnecessary head CTs as among the top five tests, procedures, and treatments to question within the ED [8]. They propose the following recommendation - don’t order CT head scans in adults and children who have suffered minor head injuries, unless positive for a validated head injury clinical decision rule [8].

The Canadian CT Head Rule (CCHR) was developed in 2001 as a framework to improve physicians’ accuracy in determining which patients that present with minor head injuries should undergo head CTs. The Canadian CT Head Rule is a clinical decision tool to help emergency physicians judiciously order head CTs for adult patients presenting with minor head injuries [9]. Since its introduction, there has been considerable interest and exploration in its application in various settings and in the impact of its use on patients and health service utilization internationally and to some extent in Canada [6]. The CCHR has been determined as the most effective of the existing clinical decision rule at minimizing testing while avoiding missed injuries in adults with minor head injuries [9]. The systematic review by Żyluk in 2015 revealed that the CCHR has a sensitivity and specificity of 100% and 48-77%, respectively [10]. The CCHR has been validated in hospitals internationally [11-13]. Despite the introduction of the CCHR in hospital systems, research has shown that it is not consistently adhered to in practice [14-16].

There is a considerable gap between our understanding of the rules of application and in the consistency of utilization of the CCHR by physicians [11-13,16]. In this study, we attempt to understand the current state of application of the CCHR in Nova Scotia. To explore this, we conducted a literature review to examine the current use of CCHR in Canada and a retrospective audit of patient charts from the Charles V. Keating Emergency and Trauma Center, an ED in Halifax, Nova Scotia. The results from this study will provide a framework for the use of the CCHR in clinical practice. In clinical practice at this site, we aim to provide data and education to improve the accurate application of the CCHR for improved patient care.

## Materials and methods

This project was reviewed by the Nova Scotia Health Ethics Review Board and deemed to be a quality study. There were two components to this work - (1) literature review and (2) chart audit.

Literature review

A narrative literature review was conducted on PubMed basic search function for “Canadian CT Head Rule,” which yielded 86 results, 62 of which were of relevance (contained the term Canadian CT Head Rule within the abstract). A separate search for “Canadian CT Head Rule adherence” was conducted, yielding 16 results, 15 of which were of relevance (contained the term Canadian CT Head Rule in the abstract). Of that 15 results, nine results were already present in the initial 62 results pulled from the search of “Canadian CT Head Rule.” In total, 68 articles were retrieved and reviewed.

Chart audit

Our primary aim was to determine if the CCHR was applied effectively in this setting. Secondary aims include presentation of data as feedback and the provision of education to increase CCHR adherence. A random sample of 25% of the patients who presented to the Emergency Department of the Charles V. Keating Emergency and Trauma Center with a head injury, facial trauma, neck trauma, or trauma between July 1, 2017, and June 30, 2018, were selected. These data were collected from our Emergency Department Information System (EDIS). We conducted a retrospective chart review of patients presenting to the Charles V. Keating Emergency and Trauma Center to assess whether the Canadian CT Head Rule was properly applied. Each chart was reviewed for desired information that was predetermined via a data collection instrument (Appendix). The data collection instrument encompassed the CCHR criteria, other relevant features of the ED visit, and details of any CT head examinations performed. The charts were reviewed by a single reviewer (AS) and data were directly entered into a database with pre-set entry fields. The reviewer (AS) collected the following listed data pertinent to the CCHR in Table 1 below.

**Table 1:** Canadian CT Head Rule. GCS: Glasgow Coma Scale

High	Medium risk
Age >65 years	Retrograde amnesia >30 minutes
Loss of consciousness	Vehicle versus pedestrian mechanism
Von Willebrand’s disease	Fall from height mechanism (>3 feet or >5 stairs)
Hemophilia	Ejected from a vehicle mechanism
Platelet disorder	-
GCS <15 at 2 hours	-
Vomiting >2 episodes	-
Depressed skull fracture	-
Antiplatelet medication	-
Anticoagulant medication	-
Battle’s sign	-
Raccoon sign	-
Hemotympanum	-

The remaining data, as follows, were pulled directly from the Emergency Department Information System (EDIS): ID, medical record number (MRN), medical services insurance (MSI), the date and time of visit, age of patient, date of birth, triage time to time seen by a physician, triage time to time of discharge, and the area within the ED that the patient was seen. The medical record number was collected to access the CT scan results and insurance number was collected in case of a typographical error in the former.

Two other reviewers (CL and SC) completed a random sample of 10% of the charts to determine Cohen’s kappa for inter-rater reliability via the following calculation: 𝜿 = (Pr{o} - Pr{e})/(1 - Pr{e}) where, Pr(o) refers to the relative observed agreement among raters, and Pr(e) refers to the hypothetical probability of a chance agreement [17]. Information was gathered from all available sources within the patient chart including the physician's note, EMS report, nursing records, and patient monitoring documentation to include all available data on the patient's condition in each case.

## Results

Review

This narrative review resulted in 62 articles, 15 of which focused on our topic specifically and are included below. The articles retrieved were grouped under broad categories - Canadian CT Head Rule (CCHR) development and context, implementation studies, and barriers to implementation and adherence.

CCHR Development and Context

Prior to its creation, a survey of members of the Canadian Association for Emergency Physicians (CAEP) on their attitudes towards different radiographic clinical decision rules revealed that 97% of physicians would consider using a CT head clinical decision rule [16]. A mathematical model revealed that use of the CCHR was the most cost-effective based on cost per quality-adjusted life years gained compared to no investigation used or sending all head injury patients for CT [18], while a review of six high-quality studies revealed that selective CT usage with the CCHR was an economically attractive strategy [19]. A retrospective review of one hospital’s unnecessary head CTs revealed that they were linked with increased costs, injury severity scores, and length of stay [20]. A prospective study comparing several head CT clinical decision tools showed that only strict adherence to the CCHR, as opposed to other tools, would result in a decrease in CT head use [21].

Implementation Studies

A review of implementation studies of radiographic clinical decision rules in the ED identified four out of five studies that showed that rule implementation effectively decreased imaging use [9]. Despite the proven effectiveness of the Canadian CT Head Rule (CCHR) in mitigating the harmful collateral effects of CT usage, its implementation is still suboptimal. Two independent studies retrospectively applied the CCHR to cases of adult patients presenting to the ED with minor head injury who received a head CT to determine the proportion of cases that were correctly referred for imaging. One study revealed that 35.3% of cases in which CT scans were ordered did not meet the CCHR [22], while the other revealed the number of unjustified cases to be as high as 61.8% [12]. A third study retrospectively applied the CCHR to all adult patients who presented to the ED with minor head injuries (not just those who were sent for CT) and found the non-compliance rate of physicians to the rule to be 28.7% [16]. A prospective study of head-injured patients that were not referred directly to the neurosurgical service and for which neurosurgery was consulted revealed that 31.2% of cases were inappropriately sent for a head CT scan [23].

Barriers to Implementation and Adherence

Various methodologies have been employed to understand the barriers to proper adherence to the CCHR. An international survey of physician awareness and use of the CCHR in four national emergency physician associations reported awareness was highest in Canada at 86% and lowest in the United States at 31% [24], while reported use was highest in Canada at 57% and lowest in the United States at 12% [25], where another rule is used primarily [26]. A survey of physicians using the Theory of Planned Behavior revealed that physicians’ attitudes are associated with intention to use CCHR, but these intentions were not significantly associated with actual CCHR use [27]. Two studies used semi-structured interviews to assess key barriers in implementing the CCHR. One used the theoretical domains framework to interview eight physicians and identified barriers in the following domains: beliefs about capabilities; behavioral regulation; memory, attention, and decision processes; environmental context and resources; and social influences [28]. The other study triangulated semi-structured interviews with cognitive task analysis and patient and physician focus groups to reveal the following key themes: patient engagement, provider confidence and experience, ability to identify and manage patient anxiety, time constraints, concussion knowledge gap, influence of health care providers, and patient expectations to get a CT [29]. Additionally, a retrospective study of CT referrals, subdivided by physician specialty, showed that 37.3% of head CTs did not follow the CCHR, with neurologists ordering the most and surgeons ordering the fewest [14]. While the CCHR was developed for the ED, overuse of CTs occurs in other specialties as well, indicating a role in developing awareness of the decision tool across all medical departments.

Data Analysis

**Figure 1:**
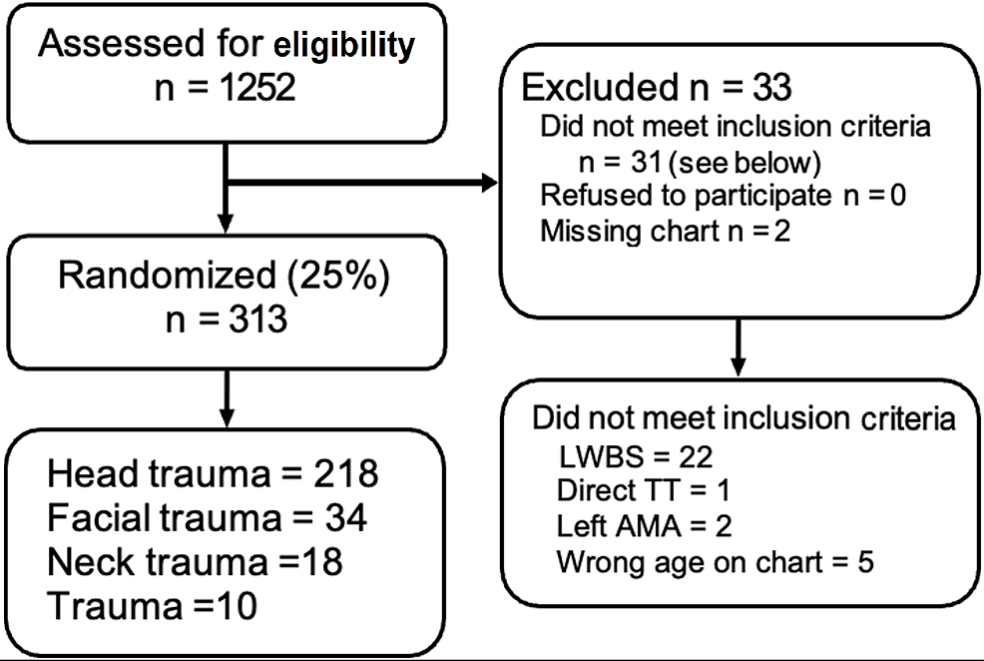
Diagram of chart selection process. LWBS: left without being seen; TT: trauma team activation; Left AMA: left against medical advice

During the study period, 1252 patients presented to the ED with one of the identified chief complaints. In total, 313 charts were reviewed for visits between July 1, 2017, and June 30, 2018. We selected 313 (25%) charts for a full review, of which 280 met our inclusion criteria. A summary of chart inclusions and exclusion is provided in Figure 1. Patients' data are outlined in Table 2.

**Table 2:** Chief complaint of included patients.

Chief complaint	Total
Head injury	218 (70%)
Facial trauma	34 (11%)
Neck trauma	18 (55%)
Trauma (general or multi-system trauma)	10 (3%)

From the total charts reviewed, when the Canadian CT Head Rule was applied, 37 (13.2%) cases met the criteria for CT indicated or CT head not indicated according to the CCHR, and 176 (62.9%) cases adhered to the CCHR CT head indications. The remaining 89 (31.8%) were excluded as the rule was not applicable. The results are summarized in Table 3.

**Table 3:** Summary of Canadian CT Head Rule (CCHR) adherence.

Adherence to Canadian CT Head Rule	CT	No CT
CT indicated	32 (86%)	5 (14%)
CT not indicated	21 (12%)	155 (88%)
Rule not applicable	24 (36%)	43 (64%)

**Figure 2:**
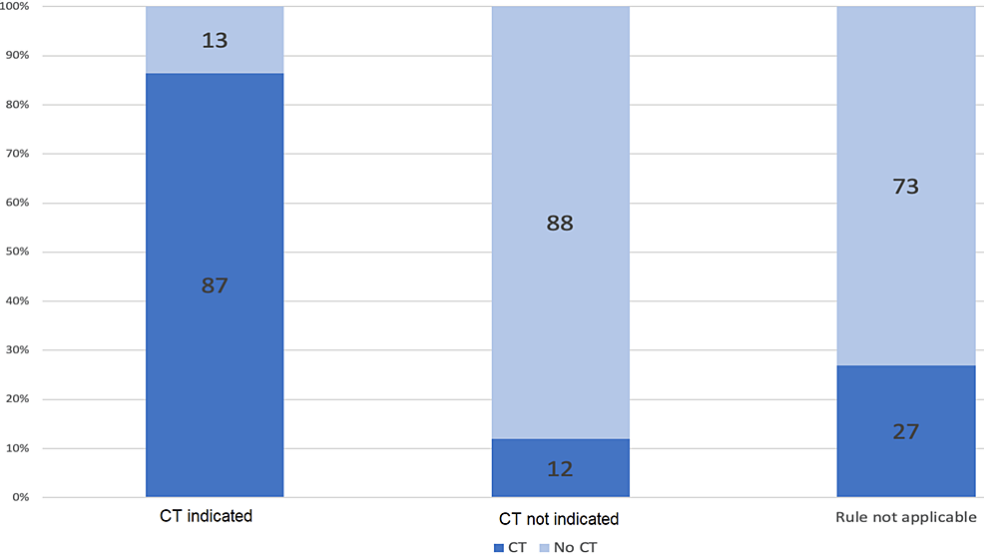
CCHR adherence as per each stratification level. CCHR: Canadian CT Head Rule

Therefore in 213 (76.0%) of the total cases, the CCHR could be applied to determine whether a CT head should be ordered. Out of the 37 cases where the CT head was indicated as per the CCHR, a CT head was ordered 32 (86.5%) times. Of the 176 cases where a CT head was not indicated as per the CCHR, a CT head was not ordered in 155 (88.1%) cases seen in Figure 2. Therefore, of the 213 cases in which the CCHR could be applied, the CCHR was followed in 187 (87.8%) cases.

Considering the cases where the CCHR was not applicable as per the exclusion criteria in Table 4, a CT was ordered in 24 (27.0%) cases as seen in Figure 2. The number of cases meeting each exclusion criteria are summarized in Table 4. The CCHR additionally stratifies its indications based on “high risk” and “medium risk” as seen in Figure 1. From our review, when a CT was indicated and ordered (total of 30), the stratification included at least one “high-risk” indicator in 18 (60%) cases and included at least one “medium-risk” indicator in 14 (46.7%) cases. When a CT was indicated but not ordered (total of 5), the stratification included at least one “high-risk” indicator in two (40%) cases and included at least one “medium-risk” indicator in three (60%) cases.

**Table 4:** Number of cases excluded. Patients can have multiple exclusions. GCS: Glasgow Coma Scale

Reasons the rule did not apply	Number of charts excluded per criteria
Patient age >65 years	4
GCS <13	9
Anticoagulant	3
Non-trauma	6
Trauma to ED time >24 hours	46
Left without being seen or against advice	22
Bleeding diathesis	0
Obvious depressed fracture	0
Pregnant	3

The outcomes of the CT scans were stratified into four categories: normal, minimal trauma (no intervention of follow-up required), minor trauma (consultation or follow-up required), and major trauma (neurosurgical intervention required). In total, 76 CTs were ordered, of which 56 were normal, nine were minimal trauma, 10 were minor trauma, and one was major trauma, as displayed in Table 4. This was then further stratified based on whether a CT was indicated, not indicated, or the rule was not applicable. We separately analyzed two potentially negative outcomes, admission to hospital and return to the emergency department within 72 hours. The results are presented based on their CCHR stratification and the outcome of whether a CT was ordered in Tables 5, 6.

**Table 5:** Cases where the patient was admitted to hospital based on CCHR stratification. CCHR: Canadian CT Head Rule

Stratification and outcome	Number of cases (percentage of cases)
CT ordered, CCHR followed	4 (13%)
CT not ordered, CCHR followed	0
CT ordered, CCHR not followed	1 (5%)
CT not ordered, CCHR not followed	0
CT ordered, rule not applicable	3 (10%)
CT not ordered, rule not applicable	2 (3%)

**Table 6:** Cases where the patient returned to the ED within 72 hours, based on CCHR stratification. CCHR: Canadian CT Head Rule

Stratification and outcome	Number of cases (percentage of cases)	
CT ordered, CCHR followed	1 (3%)	
CT not ordered, CCHR followed	4 (3%)	
CT ordered, CCHR not followed	1 (5%)	
CT not ordered, CCHR not followed	0	
CT ordered, rule not applicable	2 (8%)	
CT not ordered, rule not applicable	4 (6%)	

The 𝜿 value calculated using the equation 𝜿 = (Pr{o} - Pr{e})/(1 - Pr{e}) was 0.82 based on a review of 35 randomly selected charts (11% of the total chart sample).

## Discussion

Our care review revealed that the CCHR was adhered to in 87.8% of cases. To our knowledge, this is the first study examining adherence to the CCHR in the context of Nova Scotia Emergency Departments. This is a better rate of adherence than has been seen in the literature. Among countries where the CCHR is employed, two independent studies retrospectively applied the CCHR to cases of adult patients presenting to the ED with minor head injuries who received head CT to determine the proportion of cases that were correctly referred for imaging. One study revealed that 35.3% of cases did not meet the CCHR [22], while another revealed the number of unjustified cases to be as high as 61.8% [12]. A third study retrospectively applied the CCHR to all adult patients who presented to the ED with minor head injuries (not just those who were sent for CT) and found the non-adherence rate to the rule to be 28.7% [16]. In this study, it was found that CCHR adherence is 87.8%.

Our literature review supports the use of the CCHR as a tool that helps minimize the harms associated with unnecessary testing, improves quality-adjusted life years (QALYS), and includes other benefits such as cost-savings and decreased length of stay [18,20]. A prospective study comparing several head CT clinical decision tools found that only strict adherence to the CCHR would result in a decrease in CT head use [21]. Some of the CT scans may have been ordered to reassure patients, however, over-testing has been documented to increase patients’ level of concern more often than the opposite [30].

Although our study demonstrates a better rate of adherence to the CCHR at our institution compared with other studies in the literature, it is still not perfect. Studies suggest that there was a need for the development of the CCHR and physicians in Canada are aware of the existence of the CCHR suggesting that there is buy-in and intention to use the rule [7,8,14,19,22]. The most significant indication for a CT head scan that was missed was the use of direct-acting oral anticoagulants closely followed by patient age over 65 years despite the advanced overall age in our population. There may be several factors that contribute to lack of adherence. Our literature review suggests that intention to use the CCHR does not correlate with actual CCHR use [29]. Other barriers include systemic and individual factors, such as provider confidence, patient engagement, and environmental context and resources [28,29]. Based on these results, directions for future research could include further exploration of how to discuss the rules with patients and systemic functions that can improve adherence. 

Of note is that in 12% of patients in which a CT head was indicated no CT was performed. The indications were variable but included being on an anticoagulant or antiplatelet medication, patients whose presentations included symptomatology for which imaging is indicated based on the CCHR, and charting of symptoms present in the nursing notes but not included in the physician record. This group of patients will have to be explored in further analysis. Some potential approaches to increase adherence to the CCHR guidelines in our institution include a triage checklist for all patients presenting with head injury, adding a medication record to the triage process, the provision of regular feedback to clinicians on their documentation and adherence, and continued quality discharge instructions for all patients with head injuries.

Limitations in our study mainly stem from inherent limitations in the chart review process. Chart reviews can suffer from issues arising from both the chart creator and reviewer, including incomplete or vague chart entries, inconsistent coding of data, and inability to locate pertinent information. The paper-based chart system limited legibility and an electronic medical recording may have altered our results. Additionally, our study focussed on the practices of a single tertiary care trauma center in Nova Scotia, and therefore cannot be generalized to reflect practices across our province or across Canada.

## Conclusions

This study demonstrates that our center has good adherence to the CCHR (87.8%). The under-testing rate was equal to the over-testing indicating that without additional time or resources, better care could be offered to approximately 25% of patients despite the impressive rate of adherence.

## Appendices

**Table 7:** Data collection instrument. GCS: Glasgow Coma Scale; DOAC: direct oral anticoagulant; NOAC: non-vitamin K antagonist oral anticoagulants

Patient data	Visit features	Medical record information
Medical records number	ED disposition	High risk for intervention
Medical insurance number	Return visit within 72 hours	Loss of consciousness (minutes)
Date and time of visit	Time between ED visits (hours)	Bleeding diathesis, platelets, hemophilia, Von Willebrand’s
Age	Trauma team activation	GCS at 2 hours
Date of birth	-	Depressed or open skull fracture
Trauma to triage time (minutes)	-	Medication DOAC, NOAC anticoagulant antiplatelet
Triage to MD time (minutes)	-	Basal skull fracture sign, hemotympanum, Battle’s sign, raccoon sign
Triage to disposition time (minutes)	-	Medium risk for positive CT
ED area patient was seen	-	Amnesia before impact >30 minutes
-	-	Dangerous mechanism pedestrian in motor vehicle collision fall from a height ejected from a vehicle
-	-	Rule not applicable
-	-	GCS <13
-	-	Non-trauma
-	-	Coumadin, DOAC, bleeding diathesis
